# Determinants of Prolonged Length of Stay in the Emergency Department; a Cross-sectional Study

**Published:** 2017-01-18

**Authors:** Seyed Mohammad Hosseininejad, Hamed Aminiahidashti, Seyede Masoume Pashaei, Iraj Goli Khatir, Seyed Hosein Montazer, Farzad Bozorgi, Fahime Mahmoudi

**Affiliations:** 1 Department of Emergency Medicine, Diabetes research center, Faculty of Medicine, Mazandaran University of Medical Sciences, Sari, Iran.; 2 Department of Emergency Medicine, Faculty of Medicine, Mazandaran University of Medical Sciences, Sari, Iran.; 3 Department of Emergency Medicine, Orthopedic research center, Faculty of Medicine, Mazandaran University of Medical Sciences, Sari, Iran.; 4 Faculty of Medicine, Mazandaran University of Medical Sciences, Sari, Iran.

**Keywords:** Emergency service, hospital, length of stay, quality of health care, risk factors, Iran

## Abstract

**Introduction:**

Timeliness has been considered as a key domain in quality of emergency department (ED) care and delay in care providing is influential determinants of patient’s outcomes. The present study, aimed to evaluate the determinants of prolonged ED length of stay (LOS).

**Methods:**

In this cross-sectional study, using adopted version of the latest form for external evaluation and accreditation of EDs introduced by Iranian Ministry of Health, determinants of prolonged LOS were evaluated in the ED of an educational Hospital. Using SPSS 11, multivariate binary logistic regression was applied to estimate adjusted odds ratios (OR) for determining factors associated with prolonged LOS.

**Results:**

162 (10.2%) cases with prolonged LOS were detected. Based on univariate analysis, female gender (OR: 1.42, 95% CI: 1.14-1.75, p = 0.001), older age (OR: 1.05, 95% CI: 1.02-1.08, p < 0.0001), admission on evening shifts (OR: 4.0; 95% CI: 1.84-8.68, p < 0.001), triage level I (OR: 1.76, 95% CI: 1.21-2.57, p = 0.003), lack of insurance support (OR: 1.56, 95% CI: 1.12-2.19, p = 0.010), higher number of ordered para-clinical tests (OR: 1.23, 95% CI: 1.11-1.37, p = 0.016), and disposition time > 6 hours (OR, 0.13, p < 0.0001), were significant risk factors of prolonged LOS.

**Conclusion:**

Older age, lack of insurance support, disposition time > 6 hours due to complexity of patients’ complaint, and the necessity of repeated para-clinical measures were the most important reasons for failed provision of timely services. From the view point of ED personnel, a small part of prolonged LOS in ED was concerned with defective ED workflow, while, the most important cause of such delays was the delayed response of the consultancy services.

## Introduction

Emergency department (ED) crowding is a chronic health challenge worldwide (1). This growing challenge could results in curtailed and dysfunctional emergency activities (2, 3). It has bidirectional synergic association with delayed emergency care (2). Both crowding and delays in ED cares are influential determinants of patient’s outcome (4). Therefore, they affect ED performance, mainly regarding timeliness (5). Timeliness has been considered as a key domain in quality of emergency care. By timely we mean that EDs should continuously move toward “reducing waits and sometimes harmful delays for both those who receive and those who give care”(5). To achieve acceptable timeliness and high quality emergency care, the period from patient’s arrival to discharge could be segmented, determinants of each segment should be identified, and then evidence-based interventions may be introduced. In recent years, several interventions including employing emergency specialists, holding formal interdisciplinary team-work training programs, use of triage systems, fast-track units, and maximum length of stay (LOS) rules, e.g. 4-hour rule in the UK, have been introduced to reduce waits and delays in ED (6-9). 

In Iran, population aging has resulted in a rising number of admissions to EDs (3, 10). Accordingly, improvement of performance in EDs is an urgent challenge. Recently, a set of rules have been introduced by Iranian Ministry of Health and Medical Education (IMOH), regarding waiting times or LOS in ED, and EDs are encouraged to achieve these targets (11). In addition, Iranian researchers have focused on the LOS and shown that the average LOS of patients admitted to teaching EDs has been much more than the maximum targeted LOS (LOS<6 hours), introduced by IMOH as a safe LOS (11, 12). However, a growing body of evidence demonstrated that focusing on a set of pre-specified time rules could lead to unintended detracts from clinical priorities and, consequently, attenuation of patient-centeredness and poor outcomes (13-15).

In spite of dozens of reports on ED performance evaluation from Iran, data on the causes of lower ED performance and, specially, prolonged LOS are scarce. Therefore, this study aimed to evaluate determinants of prolonged LOS in emergency department.

## Methods

***Study design and setting***

In this cross-sectional study, using adopted version of the latest form for external evaluation and accreditation of EDs introduced by IMOH, determinants of prolonged LOS were evaluated in ED of Imam Khomeini educational Hospital, Sari, Iran, during three months from November 2014 to February 2015. Applied procedures and data collection methods in this study were approved by the ethics committee of Mazandaran University of Medical Sciences (ethical approval number: 854). Authors adhered to the all ethical principles of Helsinki declaration and confidentiality of patients’ records.

***Participants***

Patients who had stayed in the ED for more than 6 hours were considered as cases of prolonged LOS and were enrolled to the study using a sequential convenience sampling. Eligible patients were selected from the lists of all patients admitted to the ED, every 60 hours. LOS was calculated according to the following equation: 

LOS = time of medical record review - time of admission. 

Imam Khomeini Hospital is a teaching hospital affiliated to Mazandaran University of Medical Sciences. It is the most equipped hospital throughout Mazandaran province, and consequently, emergency patients are admitted to its ED, either by pre-hospital emergency teams, by themselves or their caregivers, or by other hospitals or clinics. This ED has been administered by an experienced emergency specialist and benefited from highly competent nurses and staff. According to guidelines provided by IMOH, the emergency severity index (ESI) is used for patients’ triage in this ED. 

***Data gathering***

Data gathering was done using an adopted version of the latest form for external evaluation and accreditation of EDs at teaching hospitals introduced by IMOH. The adoption process was done in a team including emergency specialists and highly competent ED staff. The first version of this checklist was used in two pilot studies and some corrections were made according to field experiences. The final version was designed in three sections including the demographic and background section, a section for indices of timely care, and a section for measuring causes of prolonged LOS. Its face validity was confirmed by a team of experts in emergency medicine. Regarding its reliability, we estimated Cronbach’s alpha as 77.0, which revealed an acceptable reliability.

The data were extracted by retrospective review of medical records, interview with the patient’s doctor, and also supervisor nurses at the time of presence of the patient in the ED. First and second sections of the study checklist were completed according to medical records, while causes of prolonged LOS were determined by in-depth interviews. Interviewees were asked to select causes of prolonged LOS of each patient according to the study checklist. A prolonged LOS could be considered owing to more than one cause. The number of prolonged LOS assigned to each cause was counted and then categorized during a review session by the research team.

Data were collected by three medical students who were trained through a series of educational sessions. During the first session, the study checklist was introduced to students and completed for selected patients. In the next role playing sessions, they were asked to complete the checklists for several complicated patients who were purposefully assigned. Then, the completed checklists were discussed and students’ competency was assessed by the principal investigator, SMH.

The data were reviewed by our principal investigator in weekly sessions. Missing and inconsistent data were specified and corrected according to medical records or ad hoc interviews, if applicable. 

***Statistical analysis***

Data were computerized and analyzed using statistical package for social sciences (SPSS) version 11.0. Data was cleaned and prepared according to recommended procedures (16) and descriptive statistics was applied to describe the data. Variable reduction was conducted using univariate statistical tests, considering P value ≤ 0.25 (17). Then, multivariate binary logistic regression was applied to estimate adjusted odds ratios (OR) and its 95% confidence intervals (CI) for associated factors with prolonged LOS. In this step, a P-value less than 0.05 was considered as statistically significant. 

## Results

***Baseline characteristics***

**Figure 1:**
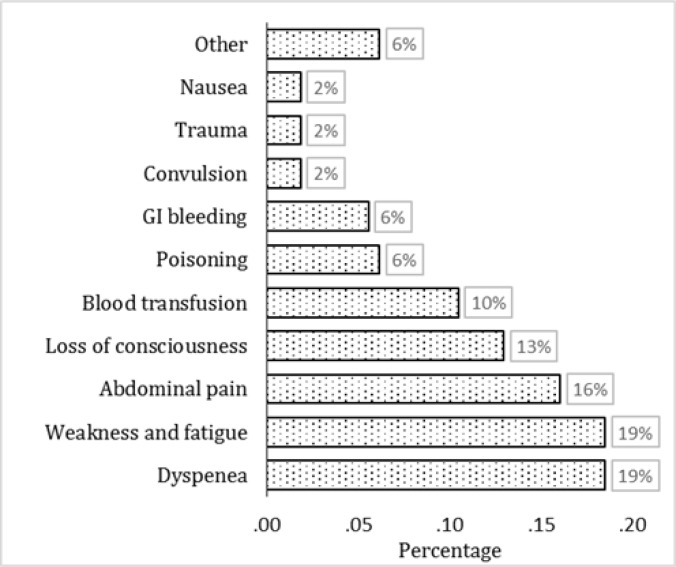
Reasons for visiting the emergency department among patients with prolonged length of stay (N = 162

1581 patients were admitted to the ED during the study period. 162 (10.2%) cases of prolonged LOS with the mean age of 58.5 ± 20.2 (Range: 12-98) years were detected (56.2% female). Table 1 shows the baseline characteristics of these 162 cases. 80.2% patients had been brought to the ED by caregivers and 78.4% had triage level of III. Figure 1 displays the reasons for visiting ED among patients with prolonged LOS. Mean waiting time from arrival to the first nursing visit was 2.85 ± 1.75 minutes (1 – 8), while mean time before the first visit by a doctor was 3.4 ± 2.7 minutes (1 – 11). 153 (94.4%) cases had disposition order within the first 6 hours of admission. 

***Determinants of prolonged LOS***

Based on univariate analysis, female gender (OR: 1.42, 95% CI: 1.14-1.75, p = 0.001), older age (OR: 1.05, 95% CI: 1.02- 1.08, p < 0.0001), admission on evening shifts (OR: 4.0; 95% CI: 1.84-8.68, p < 0.001), triage level I (OR: 1.76, 95% CI: 1.21-2.57, p = 0.003), lack of insurance support (OR: 1.56, 95% CI: 1.12-2.19, p = 0.010), higher number of ordered para-clinical tests (OR: 1.23, 95% CI: 1.11-1.37, p = 0.016), and disposition time > 6 hours (OR, 0.13, p < 0.0001), were significant risk factors of prolonged LOS. Patients had a 5% higher risk of prolonged LOS for every 5-year increase in their age. 

Based on the results of multivariate analysis, older age (p = 0.019), lack of insurance support (p = 0.024), disposition time > 6 hours (p = 0.003), and higher number of ordered para-clinical tests (p = 0.029) were significantly associated with prolonged LOS (Table 2).

***Causes of prolonged LOS based on ED personnel’s view point***

According to ED physicians and supervisor nurses, causes of prolonged LOS in these 162 cases could be categorized into ED-related factors, poor cooperation of other departments, and factors outside of the hospital (Table 3).

## Discussion

According to the findings of the present study, older age, lack of insurance support, disposition time > 6 hours, and higher number of ordered para-clinical tests were among the most important determinants of prolonged LOS. On the view point of ED personnel poor cooperation of other departments in providing proper consultancy and patients’ disposition, as well as some factors inside the ED, such as delayed consult request, complicated cases, untimely admission, and crowding, were among the most frequent causes of prolonged LOS in the studied ED. 

**Table 1:** Baseline characteristics of patients with prolonged length of stay in the emergency department

**Variable**	**N (%)**
**Gender**	
Male	71(43.8)
Female	91(56.2)
**Transferred by**	
Ambulance	130(80.2)
Caregivers	26(16.1)
Referred	6(3.7)
**Insurance support**	
Yes	141(87.0)
No	21(13.0)
**Triage level** *	
I	2(1.2)
II	7(4.3)
III	127(78.4)
IV	12(7.4)
Missing	14(8.6)
**Admission work shift**	
Night	88(54.3)
Evening	40(24.7)
Morning	34(21.0)

*: Based on emergency severity score (ESI).

**Table 2:** Determinants of prolonged length of stay in the emergency department based on multivariate analysis

**Factors**	**Crude OR **	**Adjusted OR **	**P**
Older age	1.05(1.02-1.08)	1.10(1.02-1.20)	0.019
Having insurance support	0.64(0.46-0.89)	0.29(0.10-0.85)	0.024
Disposition time ≤ 6 hours	0.13(0.02-0.99)	0.03(0.003-0.30)	0.003
Higher Number of para-clinical tests	1.23(1.11-1.37)	1.3(1.03-1.64)	0.029

Odds ratios (OR) were presented with 95% confidence interval.

**Table 3:** Sources of prolonged length of stay based on the emergency department personnel’s view point

**Factors**	**Number (%)** 1
**Inside the emergency department (n = 20)**	
Delayed request for consult	15 (75.0)
Complicated cases	5 (25.0)
Untimely admission	16 (80.0)
Crowding	2 (10.0)
**Poor cooperation of other departments** 2 **(n=114 )**	
Gastroenterology	21 (18.4)
Respiratory	12 (10.5)
Nephrology	9 (7.8)
Surgery	9 (7.8)
Orthopedic	3 (2.6)
Internal medicine	35 (30.7)
Oncology	7 (6.1)
Endocrinology	12 (10.5)
Neurosurgery	7 (6.1)
Cardiovascular	6 (5.3)
**Outside the hospital (n=28 )**	
Outside consult^3^	23 (82.1)
Imaging^4^	17 (60.7)

1 : length of stay could be prolonged due to more than one source;

2 : for patients disposition,

3 : consulting with department outside the hospital such as neurology, infectious diseases, cardiovascular, and toxicology,

4 : computed tomography scan (CT), magnetic resonance imaging (MRI), and ultrasonography.

More precisely, according to the medical and nursing staff reports, the cause of prolonged LOS, in many cases, is the delayed response by the departments with which the ED maintains interactions in order to provide proper services. Meanwhile, 6 percent of the prolonged LOS was caused as a result of defects in the ED’s workflow and ED crowding. However, this may be due to the bias of interviewees (18). Yet, considering the fact that they were asked to be honest in their statements, this bias is unlikely to be significant. 

Although, at first glance, the quantitative results differed from the causes stated by the medical and nursing staff, when we look closer, the two have largely confirmed each other. For instance, older age can increase the risk of co-morbidity and complexity of clinical decision-making (19), and may, eventually, delay the response by the consultancy services. Also, lack of insurance coverage can indicate lower socioeconomic status, increasing the risk of co-morbidity and complexity of clinical decision-making (20, 21), and eventually, delaying the response by the consultancy services. As for patients in need of repeated paraclinical measures, the consultancy services couldn’t provide proper consultation prior to receiving the respective results, hence, the delayed response by the mentioned services.

On the other hand, a part of the delayed response of the consultancy services can be associated with the reasons other than what had entered the quantitative analysis. It is possible, for instance, that defective capabilities of the staff in constructive interdisciplinary interaction (22), crowding of public medical centers while implementing the Healthcare Reform Initiative (23), technical problems of the employed equipment in paraclinical centers, and shortage of the required materials in such centers, had seriously influenced the delay of different services for timely response to the ED. It is, therefore, suggested that the reasons for delayed response of the consultancy services be examined in a study.

Our results showed that the LOS of a few patients has been prolonged due to ED crowding. This might indicate the insufficiency of the number of the staff and their adequate skills in providing timely services. That being the case, establishing a new ED in the study site is not necessary for the time being, however, designing proper interventions to obviate ED crowding can definitely prove helpful. Yet, considering the aging population in Iran (10), the effect of older age in prolonged LOS can be a warning for an increase in ED crowding (24), followed by increased ratio of patients with prolonged LOS in near future.

Although the generalizability of our results can be influenced by regional differences concerning the staff’s capabilities for interdisciplinary interaction and cooperation (25), regional infrastructures and development, population care patterns, and patient distribution, but since Sari, is among the fairly developed Iranian cities, we hope that the results of this study are generalizable to most Iranian EDs, especially those situated in areas with populations below one million. In any case, further studies can reveal the generalizability of our findings.

***Limitation***

Unfortunately, we couldn’t manage to conduct prospective follow-up of the patients admitted to the ED to determine the outcome of patients with prolonged LOS, as well as, their distribution by time of discharge. Considering the conditions of the patients and also the nature and limitations of the study, we failed to contact the patients to collect more data. We also couldn’t examine, with adequate accuracy, the contributing factors and causes of disposition after 6 hours from admission. We believe these limitations can be objectives for further studies.

## Conclusion:

Older age, lack of insurance support, disposition time > 6 hours due to complexity of patients’ complaint, and the necessity of repeated para-clinical measures were the most important reasons for failed provision of timely services. On the view point of ED personnel, a small part of prolong ED length of stay was concerned with defective ED workflow, while, the most important cause of such delays was the delayed response of the consultancy services.
